# Graph Based Study of Allergen Cross-Reactivity of Plant Lipid Transfer Proteins (LTPs) Using Microarray in a Multicenter Study

**DOI:** 10.1371/journal.pone.0050799

**Published:** 2012-12-14

**Authors:** Arantxa Palacín, Cristina Gómez-Casado, Luis A. Rivas, Jacobo Aguirre, Leticia Tordesillas, Joan Bartra, Carlos Blanco, Teresa Carrillo, Javier Cuesta-Herranz, Consolación de Frutos, Genoveva García Álvarez-Eire, Francisco J. Fernández, Pedro Gamboa, Rosa Muñoz, Rosa Sánchez-Monge, Sofía Sirvent, María J. Torres, Susana Varela-Losada, Rosalía Rodríguez, Victor Parro, Miguel Blanca, Gabriel Salcedo, Araceli Díaz-Perales

**Affiliations:** 1 Centro de Biotecnología y Genómica de Plantas (UPM-INIA), Campus de Montegancedo, Pozuelo de Alarcón, Madrid, Spain; 2 Departamento de Evolución Molecular, Centro de Astrobiología (INTA-CSIC), Torrejón de Ardoz, Madrid, Spain; 3 Unitat d'Alèrgia Servei Pneumologia i Alèrgia Respiratòria Universitat de Barcelona, Institut d'Investigacions Biomèdiques August Pi i Sunyer (IDIBAPS), Centro de Investigaciones Biomédicas en Red de Enfermedades Respiratorias (CIBERES), Barcelona, Spain; 4 Servicio de Alergia, Hospital Universitario de la Princesa, Instituto de Investigación Sanitaria Princesa (IP), Madrid, Spain; 5 Servicio de Alergología, Hospital Universitario de Gran Canaria Dr. Negrín, Las Palmas de Gran Canaria, Spain; 6 Unidad Servicio de Alergia, Fundación Jiménez Díaz, Madrid, Spain; 7 Allergy Section, General University Hospital, Miguel Hernández University, Elche, Spain; 8 Unidad de Alergología, Complexo Hospitalario, Ourense, Spain; 9 Research Laboratory, Fundación IMABIS-Carlos Haya Hospital; Hospital Civil, Málaga, Spain; 10 Servicio de Alergia, Hospital de Basurto, Bilbao, Spain; 11 Departamento de Bioquímica y Biología Molecular I, Facultad de Químicas, Universidad Complutense, Madrid, Spain; 12 Departamento de Biotecnología, ETSI Agrónomos, Universidad Politécnica, Madrid, Spain; Murdoch University, Australia

## Abstract

The study of cross-reactivity in allergy is key to both understanding. the allergic response of many patients and providing them with a rational treatment In the present study, protein microarrays and a co-sensitization graph approach were used in conjunction with an allergen microarray immunoassay. This enabled us to include a wide number of proteins and a large number of patients, and to study sensitization profiles among members of the LTP family. Fourteen LTPs from the most frequent plant food-induced allergies in the geographical area studied were printed into a microarray specifically designed for this research. 212 patients with fruit allergy and 117 food-tolerant pollen allergic subjects were recruited from seven regions of Spain with different pollen profiles, and their sera were tested with allergen microarray. This approach has proven itself to be a good tool to study cross-reactivity between members of LTP family, and could become a useful strategy to analyze other families of allergens.

## Introduction

The fact that allergic patients sensitized to a particular allergen can exhibit an allergic response to other agents shows that the same IgE antibodies are able to recognize homologous allergens from different species that share the same epitopes. This phenomenon is known as cross-reactivity [1] and is a major concern in allergy. The cross-reactivity allergens share the same epitopes but can differ mainly in the identity of their primary sequences and often in their three-dimensional structure. Understanding the factors and identifying the number of allergenic sources that are involved in cross-reactivity are essential for improving the diagnosis and treatment of allergic diseases [2], [3]. To address these issues, a large number of allergens of the same family and sera from a large number of patients are needed because of the heterogeneity of patients' responses. These requirements make the study of cross-reactivity using classical approaches difficult [4], [5] and require the use of higher capacity technologies.

The introduction of microarray techniques featuring a large panel of purified allergens has been a major advance in the diagnosis of allergic diseases [6], [7], [8]. Simultaneous measurements of IgEs specific to many proteins from the same family using minimal quantity of allergen and sera are now possible, allowing many samples to be screened at reasonable cost. The huge amount of information generated by these microarrays also demands more powerful analytical strategies to identify associations within the obtained data [5], [6], [7], [8], [9], [10].

In the context of graph theory, graphs, also known as networks [7], [10], represent relationships between objects and have been used to obtain reliable information from many different biological systems [11]–[16], including a network that has been established for human diseases [17]. In particular, the graph theory has recently been used to describe cross-reactions between antibodies in a sandwich microarray immunoassay [17], and we use it in a similar way here to study allergen cross-reactivity.

The family of plant lipid transfer proteins (LTPs) has been widely studied with respect to plant food allergenic reactions and their role in food and/or pollen cross-reactivities. Allergens from this family have been found in most vegetable sources. Pru p 3, the peach LTP, is the model member of this family. It is recognized by 75% of patients who suffer from peach allergy, the most frequent plant food allergy in Spain [18], [19]. Pru p 3 has been implicated in food cross-reactivities, especially those involving fruits and nuts, and pollens such as those of mugwort and plane [20], [21].

The principal aim of this study was to study cross-reactivity in plant foods using as models the LTP family and their recognition by fruit-allergic patients from several regions of Spain, taking into account the different pollen profiles in these areas. For this purpose we used competitive cross-inhibition immunoassay and the analytical potential of the theory of graphs.

## Results

### Cross-reactivity between LTPs

**Figure 1:**
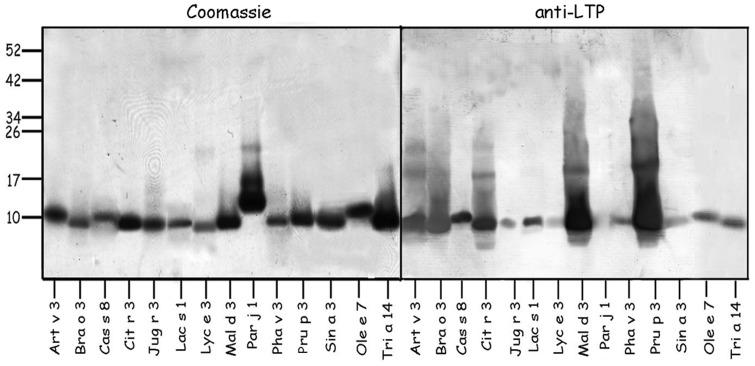
Lipid transfer proteins (LTP) included in the homemade array were separated by SDS-PAGE and stained with Coomassie Blue. Replicas were electrotransferred and incubated with polyclonal antibodies produced against peach LTP (dilution 1∶500). The name of the proteins corresponds to Table I.

**Figure 2:**
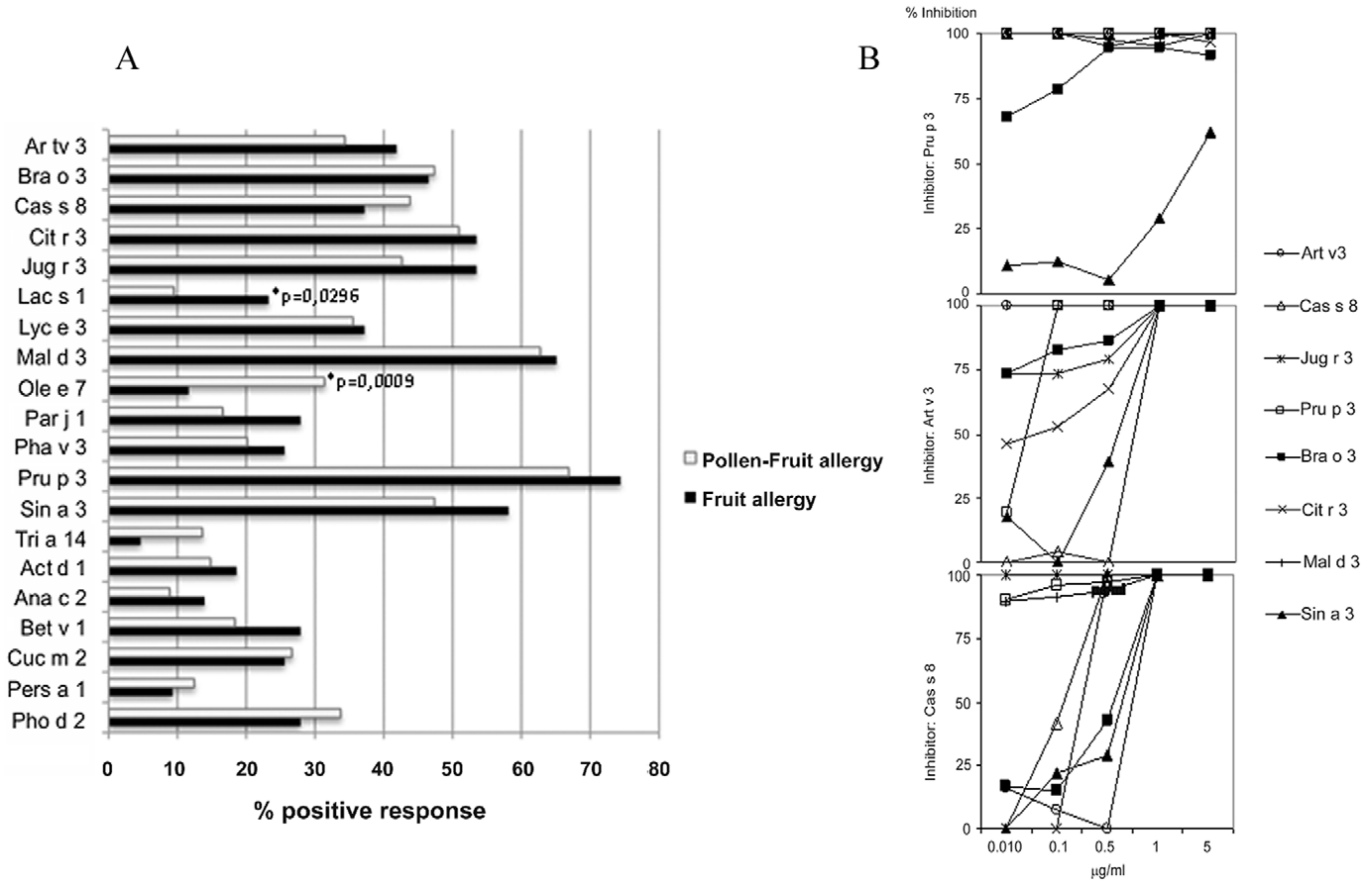
Frequency of LTP sensitization. **A**. Recognition frequencies of food and pollen LTPs comparing fruit allergic patients with and without pollen sensitization (Pollen Fruit Allergy and Fruit Allergy, respectively). The recognition frequencies, shown as percentage of positive response (%), were obtained incubating the LTP microarray with single sera from allergic patients. **B**. Analysis of LTP crossreactivities by inhibition assays using the LTP microarray as solid phase and Pru p 3, Art v 3 and Cas s 8 (5, 1, 0.1, 0.01 µg/mL) as inhibitors. The inhibition percentage of the IgE binding capacity is indicated. Means (n = 3) and SDs (bars) are represented. All tests were performed in triplicate.

Considering the allergies most frequently associated with peach sensitization in Spain [19], [22], [23], 14 LTPs from those foods and pollens relevant to the study population (Table 1; see Materials and Methods for details) were selected to construct a homemade microarray (LTP microarray). All of the allergens were tested by Western-blot with a polyclonal antibody against Pru p 3 (peach LTP; 20) (Figure 1) as well as by N-terminal amino acid-sequencing and mass spectrometry (Table 1). A comparison of the group of fruit allergic patients with food-tolerant pollen allergic subjects (Table 2) confirmed an association between LTP sensitization and fruit allergy (Figure 2A). By contrast, LTP frequencies were very similar in the group of fruit allergic patients, regardless of their inhalant sensitization profiles.

**Table 1:** Purified proteins included in the LTP microarray.

Protein	Family	Species/Tissue	N-terminal or internal peptide sequence	Accession number	Reference
**Art v 3**	LTP	*Artemisia vulgaris*/pollen	ALTXSDV	P0C088	[20]
**Bra o 3**	LTP	*Brassica oleracea*/leaf	AISXGTVTSNLAPXAVYLMK	Q9ZSL7	[31]
**Cas s 8**	LTP	*Castanea sativa*/fruit	SITXTQVSKLMPXL	Q42952	[20]
**Cit r 3**	LTP	*Citrus reticulata*/fruit	ITXGQVTGSLAPCIAFRLTG	P84161	[32]
**Jug r 3**	LTP	*Juglans regia*/fruit	VITXGQVASS	C5H617	[33]
**Lac s 1**	LTP	*Lactuca sativa*/leaf	AISXGQVTANLAGXL	A1E2H5	[34]
**Lyc e 3**	LTP	*Lycopersicon esculentum*/fruit	AITXGQVDAN	P27056	[35]
**rMal d 3**	LTP	*Malus domestica*/fruit	EITCGTV	Q9M5X7	[36]
**Ole e 7**	LTP	*Olea europaea*/pollen	APSQSTVTALLTSCVSYIDDQ	P81430	[29]
**rPar j 1**	LTP	*Parietaria judaica*/pollen	AGLAWTSLASVAP	P43217	Commercial
**Pha v 3**	LTP	*Phaseolus vulgaris*/fruit	K.QLSASVPGVNANNAAALPGK.C K.CGVNVPYK.IKQLSASVPGVNANNAAALPGKC KCGVNVPYKI	Q9M5X8 (O24440)	[37]
**rPru p 3**	LTP	*Prunus persica*/fruit	ITCGQE	Q9LED1	[36]
**rSin a 3**	LTP	*Sinapis alba*/seed	ALSXG	EF626938	[38]
**rTri a 14**	LTP	*Triticum aestivum*/wheat	IDCGHVD	AJ852536	[28]
* **Act d 1**	Cysteine protease	*Actinidia deliciosa*/fruit	LPSYV	P00785	[39]
* **Ana c 2**	Cysteine protease	*Ananas comosus/pineapple* commercial	MAEYGRVYKDNDE	BAA21929	Commercial
* **Bet v 1**	PR10	*Betula verrucosa*/pollen	ARLFKAFILDGDNL	P15494	Commercial
* **Cuc m 2**	Profilin	*Cucumis melon*/fruit	MSWGAYVDDHLMC	AJ565931	[40]
* **Pers a 1**	Class I chitinase	*Persea americana*/fruit	EQHGR	P93680	[41]
* **Pho d 2**	Profilin	*Phoenix dactylifera*/pollen	MSWGAYVDEHLMC	AJ417566	Commercial

r recombinant protein;

* non-LTPs or control proteins included in the array are marked with an asterisk.

**Table 2:** Clinical data of patients included in this study.

	Fruit allergic patients	Pollen-food tolerant allergic patients
Number of patients	212	117
**Allergy Symptoms (%)**		
Rhinitis	76	87
Asthma	27	43
Oral allergy syndrome	42	0
Anaphylactic reaction	14	0
Urticaria	50	2
Angio-edema	13	0
Gastrointestinal	7	0
Others	3	0
**SPT (%)^3^**		
Mugwort	40	45
Cypress	22	31
Grass	40	88
Plane	42	31
Olive	43	39
Pellitory	4	13

As expected, the most prevalent LTP was Pru p 3, which was slightly more frequently recognized by fruit-allergic patients without pollen allergy than by those who also had respiratory diseases, although these differences (74% versus 67%; Figure 2A) were not statistically significant. This tendency was observed for other allergens such as Sin a 3 (58% versus 47%) and Jug r 3 (53% versus 43%).

Cross-reactivity between LTPs was demonstrated by inhibition assays, selecting three allergens as models: Pru p 3 (from fruit), Art v 3 (from pollen) and Cas s 8 (from nuts) (Figure 2B). The peach LTP, Pru p 3, was able to completely inhibit the IgE binding of printed LTPs, with the exception of Sin a 3. Cas s 8 and Art v 3 were more specific in their inhibition, although both were also able to inhibit the IgE binding of the other. These data suggest that Pru p 3 acts as the principal primary sensitizer of these types of allergies and as the main gateway to patients' poly-sensitization to LTPs.

### Graph-based analysis of the LTP microarray immunoassay

**Figure 3:**
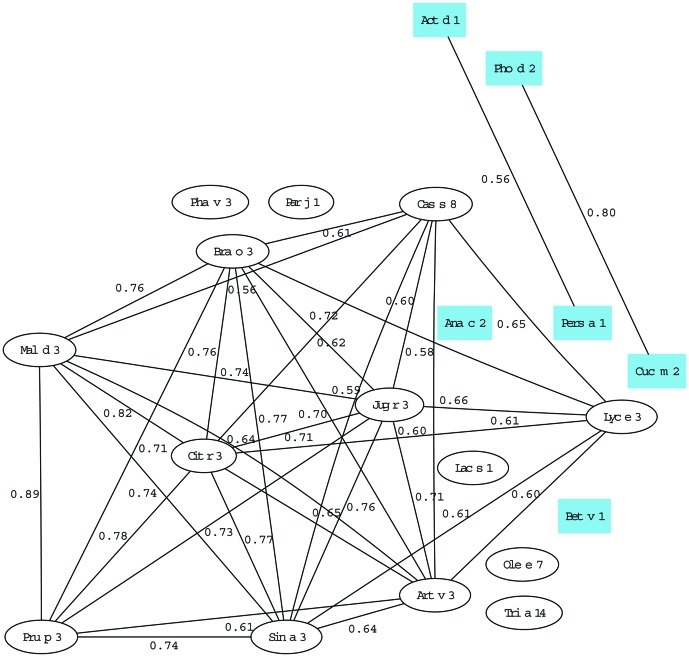
Co-sensitization graph of LTP allergens. Each node represents one allergen (LTP, white ovals non-LTP allergens, blue squares) and the links represent co-sensitization of one or more sera for the linked allergens. The weight of each link, between 0 and 1, measures the degree of co-sensitization. For the sake of clarity, only the 35 links of weights greater than 0.55 of the total 190 existing links are plotted.

In this study, graph theory has been used not only as a way to represent LTP-microarray immunoassay data, but also to gain insight into co-sensitization patterns of the LTP allergens in the selected allergic population. The construction of the co-sensitization graph associated to our LTP microarray immunoassay, shown in Figure 3, is explained in detail in *Materials and methods*. In summary, two allergens are connected by a link of the graph if at least one serum gave a positive reaction to both allergens. The weight of such a link is a measure of the degree of similarity between the sera that reacted positively to each of the allergens; the maximum weight of 1 is obtained when both allergens were recognized by exactly the same group of reacting sera (irrespective of the size of the group).

In this way, we were able to confirm that our co-sensitization graph was totally connected (i.e., all allergens were interconnected). This means that at least one serum reacted positively to any given pair of allergens, that is, there were no incompatible pairs of co-sensitizations. Therefore, there are (20×19)/2 = 190 links in the graph, although in order to clarify the interpretation of results only the 35 with a weight greater than 0.55 are plotted in Figure 3. The allergens connected with the highest weights, i.e., those undergoing the strongest co-sensitization, were Pru p 3 and Mal d 3 (0.89), Mal d 3 and Cit r 3 (0.82) and the non-LTP allergens Cuc m 2 and Pho d 2 (0.80). As expected, the lowest co-sensitizations were found between LTP allergens and non-LTP allergens: Pers a 1 and Tri a 14 (0.19) and Ana c 2 and Ole e 7 (0.19). The lowest co-sensitizations between two LTP allergens were found in Par j 1 and Lyc e 3 (0.29) and Par j 1 and Sin a 3 (0.31).

The average weights shown in Table 3 and mathematically defined in M*aterials and methods* are a measure of the average degree of co-sensitization that an allergen showed with the other allergens in the graph. As expected, Pru p 3 had a very high average weight of 0.49, meaning that it is one of the most robustly connected nodes of the network. Bra o 3, Cit r 3 and Mal d 3 had the highest average weights and were all above 0.5, while the non-LTP allergens had very low average weights (all less than 0.37). The case of the non-LTP allergens Cuc m 2 and Pho d 2 is amazing, since these allergens are very highly co-sensitized because they belong to the same protein family (the profilins), but their average weight was extremely low because, as non-LTP allergens, their co-sensitizations with the rest of the nodes were very low. Finally, it is useful to highlight attention the case of Par j 1 and Tri a 14, the LTP-allergens with the lowest average weight (0.36 and 0.34, respectively).

**Table 3:** Average weight of allergens included in the co-sensitization graph.

Protein	Family	Average weight
Bra o 3	LTP	0.52
Cit r 3	LTP	0.51
Mal d 3	LTP	0.50
Jug r 3	LTP	0.50
Art v 3	LTP	0.50
Sin a 3	LTP	0.49
Pru p 3	LTP	0.49
Cas s 8	LTP	0.48
Lyc e 3	LTP	0.46
Lac s 1	LTP	0.40
Pha v 3	LTP	0.40
Pho d 2	Profilin	0.38
Ole e 7	LTP	0.37
Cuc m 2	Profilin	0.37
Par j 1	LTP	0.36
Act d 1	Cysteine protease	0.35
Tri a 14	LTP	0.34
Bet v 1	PR10	0.32
Ana c 2	Cysteine protease	0.31
Pers a 1	Class I chitinase	0.30

### Pattern of responses by geographical area

Subjects with fruit allergy and without any inhalant symptoms tended to recognize more LTPs (α ¯ = 6) than those who were sensitized to fruit and pollen (α ¯ = 5), although the difference was not significant (Mann-Whitney U test: *p* = 0.0561). Curiously, individuals from Barcelona and the Canary Islands showed a high degree of polysensitization (α ¯ = 9 and α ¯ = 7 LTPs, respectively; Mann-Whitney test: p = 0.0010). This was markedly higher than those from Ourense (α ¯ = 3), who exhibited the lowest frequencies for most of the LTPs analyzed.

**Figure 4:**
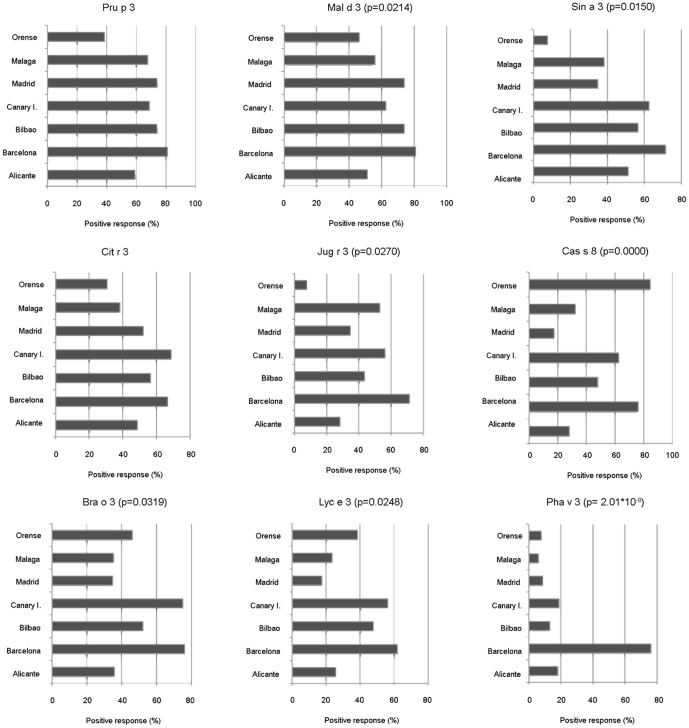
Recognition frequencies of food and pollen LTPs by geographical area. The recognition frequencies, shown as percentage of positive response (%), were obtained incubating the LTP microarray with single sera from allergic patients. Only LTPs with a positive response of more than 20% (taking all patients into account) are represented. Percentage positive responses and significant differences (p<0.05) are indicated.

The frequencies of recognition in most areas for Pru p 3 were very similar, except in Ourense, where fewer than 40% of patients recognized Pru p 3 (Figure 4). In this area, Cas s 8, the chestnut LTP, was the most prevalent LTP with recognition of more than 80%. Considerable consumption of chestnut is typical in this area, where patients suffer from pollen and nut allergy. With respect to the other nut LTP, Jug r 3, people from the Canary Islands, Barcelona and Málaga, but not subjects from Madrid, Bilbao or Alicante, exhibited the highest recognition frequencies (Figure 4).

Of the pollen LTPs included in this study, only Art v 3 was clearly associated with fruit sensitization (Figure 3; χ^2^ = 0.001). Areas such as Barcelona, the Canary Islands and Bilbao had the highest frequencies of this allergen (>50%). The high frequency in the Canary Islands could be due to the presence of this pollen, but cross-reactivity may explain the high degree of recognition of this pollen allergen (40%) by patients from Barcelona and Bilbao.

## Discussion

The analysis of the IgE binding of members of the same allergenic family using data from large numbers of patients could clarify recognition patterns and help us to predict cross-reactivity in food allergy. In order to study plant food cross-reactivity, a representative panel of LTP allergens was produced, based on the most frequent sensitizations associated with plant food allergy in our area [19], [22], [23]. The quantity of proteins required and the number of patients led us to adopt the microarray approach to ensure the accuracy of our study. Analysis of sensitization profiles for allergies using microarray strategies is a powerful method, and several research groups have obtained relevant results with this technique [7], [8], [9], [24]–[26] for a variety of pathologies. It can help us to improve the diagnosis and treatment of allergy because many allergens can be tested in a large number of patients in the same assay. The lower cost and the more rapid processing of samples are also advantages [5], [7].

Lipid transfer proteins have been classified as important food allergens and are often involved in many plant food and food/pollen cross-reactivities [27]. In our study, Pru p 3 yielded the highest recognition frequencies, followed by Mal d 3, Cit r 3, Bra o 3 and Sin a 3 LTP (all of them around 50%). The nut LTPs, Jug r 3 and Cas s 8, also had a very high prevalence (>40%). These values were consistent with previously published results concerning one of the most frequent cross-reactivities with peach observed in the routine clinical milieu [19], [22], [23]. By contrast, Tri a 14, the wheat LTP responsible for baker's asthma [28] was seldom recognized in the population that we studied. The low response to Tri a 14 suggests that this LTP is of no relevance in fruit allergy.

With respect to inhaled LTPs, Art v 3 was the only one clearly associated with fruit allergy, in accordance with previously described results [20]. However, the other pollen allergens, Ole e 7 and Par j 1, gave very different recognition patterns from that of Pru p 3. The lack of any relation between Ole e 7/Par j 1 sensitization and peach allergy has recently been described [29].

The study of LTP cross-reactivity by IgE binding inhibition suggested that Pru p 3 was the primary sensitizing in the majority of reactions, but not the only one. Pru p 3 was not able to fully inhibit IgE binding of Sin a 3, or mustard LTP. This peculiarity implies that the mustard LTP includes specific epitopes, being other gateway into the syndrome of LTPs.

The fruit-allergic patients recognizing the largest number of LTPs came from Barcelona and the Canary Islands. The presence of cross-reactive pollen LTPs such as Pla a 3, from plane pollen, and Art v 3, from mugwort pollen [20], [21], may increase the polysensitization to this allergenic family. The pollen season is annual and may favour constant LTP sensitizations and, unlike food, it is unavoidable.

The graph analysis also revealed strong associations between allergens. Pru p 3 presented very high co-sensitizations with many other allergens, suggesting that a fruit-allergic patient has a high probability of being sensitive in turn to apple, cabbage, nut, mustard and tangerine. However, it seems that subjects do not easily develop allergy against green beans or lettuce in the first stage of polysensitization. All these results are known in routine clinical circumstances but they represent the first experimental evidence of the syndrome of LTPs with a large number of allergens. Therefore, our model is valuable due to its ability to illustrate i) the degree of cross-reactivity between LTP members and ii) the probability that patients allergic to a given LTP will be sensitized to others. These results open up the possibility of analyzing other routes of sensitization obtained from the graph analysis, and therefore represent a promising line of future work.

In summary, the array approach and the application of graph theory have proved to be useful tools in this type of study. Fruit-allergic patients from Spain were usually polysensitized to multiple members of the LTP family, with the sensitizations to peach, apple, tangerine, cabbage, mustard and nuts being the most noteworthy. LTP recognition profiles may be influenced by local pollen patterns, especially by some pollen LTPs such as mugwort and plane allergens.

## Materials and Methods

### Selection and purification of allergens

Thirteen LTPs were purified by Diaz Perales' and Rodriguez's laboratories following a previously published method from natural source or as recombinant protein in the yeast *Pichia pastoris* (rMal d 3, rPru p 3, rSin a 3, rTri a 14), and one, Par j 1, was produced as recombinant in *Escherichia coli* by Bial Aristegui (Bilbao, Spain) [19]–[21], [28]. These proteins were chosen on the basis of their previously described allergenic properties and their relationship with peach allergy. Additional allergens were obtained to complete the allergen array: Pru p 2.0102 (TLP, peach allergen), Act d 1 (cysteine protease, kiwi allergen), Ana c 2 (pineapple allergen), Bet v 1 (PR10 from birch pollen), Cuc m 2 (melon fruit profilin), Pers a 1 (avocado latex-fruit allergen) and Pho d 2 (palm-pollen profilin).

All purified proteins were identified by trypsin peptide- and/or N-terminal amino acid-sequencing and mass spectrometry (MALDI-TOF-TOF Autoflex, Bruker, Bremen, Germany) in the Proteomic Service of CIB, CSIC (Madrid, Spain). In addition, every purified protein (5 µg) was visualized on Coomassie-stained SDS-PAGE and by incubation with rabbit polyclonal antibodies to Pru p 3 (1∶500 dilution). Protein preparations were considered to contain homogeneous molecules after adequate verification to rule out the possibility of contamination.

### Characteristics of the allergic population included

Patients from seven different regions of Spain were selected following the same criteria. The regions were chosen based on the regions with the highest pollen counts (Table 4, pollen count average of the last 10 years). Fruit-allergic patients (n = 212) were included in the study, prospectively among the adult population (Table 2). Criteria for inclusion were: i) a consistent history of adverse reaction to fruits, indicative of IgE-mediated allergy and ii) giving positive results to the skin-prick test and open food challenge, following the diagnostic algorithm recommended by official allergy academies [30]. SPT responses were performed following EAACI recommendations [30]. The Ethics Committee of each hospital approved the study: the Ethic Committee of Hospital Clinic de Barcelona; the Ethic Committee of Hospital Universitario de la Princesa; the Ethic Committee of Hospital Universitario de Gran Canaria Dr. Negrín; the Ethic Committee of Fundación Jiménez Díaz; the Hospital General de Alicante; the Ethic Committee of Complexo Hospitalario de Ourense the Ethic Committee of Hospital Civil, Málaga; the Ethic Committee of Hospital de Basurto, Bilbao; the Ethic Committee of Universidad Politécnica de Madrid (Spain). Patients and control volunteers also gave their written informed consent to their participation.

**Table 4:** Pollen counts (grains/m^3^ of air) of the regions included in the study.

Region Pollen*	Alicante (Elche) (2001–2011)	Barcelona (2001–2011)	Bilbao (2001–2011)	Canary Islands (2008–2011)	Madrid (2001–2011)	Málaga (1995–1999)	Ourense (1996–1999)
**Grass**	-	-	-	-	200	-	-
**Birch**	-	-	100	-	-	-	120
**Plane**	-	1800	-	-	1050	225	-
**Oak**	-	375	330	-	520	-	100
**Pine**	375	200	620	100	160	-	130
**Cypress**	175	525	-	-	200	250	470
**Mugwort**	-	-	-	150	-	-	-
**Pellitory**	-	-	-	110	-	-	-
**Olive**	355	125	-	-	473	1250	-
**Palm**	220	-		-	-	-	-

* Average pollen counts (grains/m^3^). The period is indicated in brackets.

Data were obtained as the average of the previous years (period in parentheses), from the Comité de Aerobiología-SEAIC (http://www.polenes.com/concentraciones.html) and the PIA-Punto de información de Aerobiología-UAB (http://lap.uab.cat/aerobiologia/).

Patients with seasonal rhinitis and food tolerance were included as a control group with the following criteria: a compatible clinical history of pollinosis confirmed by positive skin-prick tests to pollen allergens, but without any symptoms of plant food allergy and negative responses to food extracts by SPTs (n = 117). These patients showed mainly positive responses to mugwort, olive and grass pollen (Table 2). An additional group of food-tolerant volunteers without pollinosis (n = 35; 5 per region) was recruited as a negative control. Most of them (n = 27) were atopic suffering from dust mite and animal dander allergies.

### Production of allergen microarray and immunoassay

Purified proteins (0.25 mg/ml and 0.125 mg/ml in 1X Protein Binding Buffer (Whatman, USA) containing 0.02% Tween 20) were applied on epoxy-activated glass slides (TeleChem International, Sunnyvale, CA, USA) with 16 microarrays per slide, using a MicroGrid II TAS arrayer (BioRobotics, Genomic Solutions, Ann Arbor, MI, USA). Several protein concentrations (1, 0.75, 0.5, 0.25 and 0.125 mg/ml) were tested and those that resolved best were chosen (data not shown). Labelled pre-immune antibody was spotted as a guide dot to support automatic image analysis. Gaskets (TeleChem International, Sunnyvale, CA, USA) were attached to the slides to create a barrier between the 16 arrays and sealed to prevent evaporation. Each array well was incubated for 1 hour at room temperature with blocking solution (Sigma, St. Louis, CO, USA) and then incubated overnight with 80 ul undiluted serum at 4°C. To detect bound IgE antibodies, the slides were incubated for 1 hour at room temperature with anti-human IgE labelled with PE-DY 647 (Thermo Scientific, Rockford, IL, USA) diluted 1∶100. As a blank control, one array well per slide was always incubated with PBS alone (Sigma, St. Louis, CO, USA) instead of serum, and after washing, incubated with the fluorescence secondary antibody. PBS containing 0.1% Tween 20 was used as washing solution. Three spots from the same sample were included in each array, and two replicates of each assay were performed (Pearson correlation = 0.83; *p*<0.0001).

Spots with obvious defects and those replicate spots with a signal-to-noise ratio less than 3, as measured by GenePix™ software (Genomics Solutions, PE, USA), were removed from the analysis. Only those allergen spots with at least two to three replicates fulfilling the analysis criteria were considered for quantification. The IgE binding of each allergen spot was calculated as the final fluorescence intensity, obtained by subtracting the local background *B* from the observed value, measured by GenePix™ software and then the fluorescence intensity from the blank control by applying the equation: *I* = (F_645_ – *B*)_sample_ – (F_645_ – *B*)_blank_. Fluorescence intensity levels >200 units were considered to be positive (highest value of mean +3× SD of negative control spots, those containing only blocking solution).

### Immunoinhibition assay on the LTP array

The inhibition assays were carried out as explained in the former section for immunoassays, but with the following modifications. A pool of sera (n = 20 fruit allergic patients) was incubated with increasing amounts of LTPs used as inhibitors (0.01, 0.1, 0.5, 1, 5 mg/mL) for 3 hours at room temperature: Pru p 3, as fruit LTP; Cas s 8, as nut LTP; and Art v 3, as pollen LTP. The preincubated pool was then added to the LTP array blocked with blocking solution. All tests were performed in triplicate. The inhibition (%) of IgE binding was determined as:
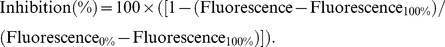
Fluorescence stands for the result of the immuno-inhibition incubating the pool of sera with LTP inhibitor, Fluorescence_0%_ stands for the result incubating the pool of sera without LTP inhibitor, and Fluorescence_100%_ stands for the result incubating LTP inhibitor in the absence of the pool of sera.

### Construction of the co-sensitization graph associated with an LTP microarray immunoassay

A weighted and undirected co-sensitization graph ***G_A_*** can be associated with an LTP microarray immunoassay to study the co-sensitization between LTPs as follows. The elements of the matrix of fluorescence intensities ***I*** are first defined as the fluorescence *I_ij_* obtained when the serum IgE *i* binds to the allergen *j*. Therefore, each column of ***I*** represents the average of two microarray immunoassays in which the same patient serum is incubated on the LTP-microarray and revealed with fluorescently labelled anti-human IgE (see former subsection). We call ***B*** the matrix defined by *B_ij_* = 1 when *A_ij_>0*; otherwise, *B_ij_* = 0.

Graphs are bipartite when they have two or more types of nodes, and links only connect nodes of different type. Graph ***B*** associated with matrix ***B*** is a bipartite graph. Nodes of type S represent the *N_S_* = 329 sera and nodes of type A represent the *N_A_* = 20 allergens (14 LTPs and 6 non-LTP allergens). Two nodes, *i* (serum) and *j* (allergen), are connected in the bipartite graph ***B*** if the value *B_ij_* = 1, which means that subject *i* has given a positive allergic reaction to the allergen *j*.

We then project the bipartite graph ***B*** onto graph ***G_A_***, a graph with nodes of only one type (allergens). We call it the co-sensitization graph and visualize it using the open source graph visualization software GRAPHVIZ (tool NEATO). The links in graph ***G_A_*** connect two allergens *m* and *n* if, in the original bipartite graph ***B***, these two allergens were connected to one or more common sera. The weight *w_m,n_* of such a link between *m* and *n* takes values from 0 to 1 and measures the *similarity* between the neighbors of allergens *m* and *n* in the bipartite graph. We use the cosine distance between two vectors to calculate *w_m,n_*:
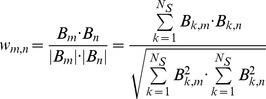
where *B_m_* and *B_n_* are the *m*- and *n*- columns of matrix *B*, respectively. Therefore, the weight *w_m,n_* is zero, and there is no link between *m* and *n*, when *B_m_* and *B_n_* have no common elements (that is, when not even one serum has a positive reaction to both allergens *m* and *n*), while *w_m,n_* reaches its maximum value of one when both vectors are identical (that is, when allergens *m* and *n* are recognized by the same group of reacting sera).

Finally, the average weight of a node is calculated as:
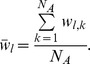
The average weight of an allergen *l* measures the average value of the co-sensitizations between *l* and all other allergens represented in the graph.

### Statistical analysis

Fluorescence levels (in arbitrary units) obtained from each patient's serum were analyzed using contingency tests. Differences in the quantitative variables were analyzed by the non-parametric Mann-Whitney U and Kruskal-Wallis tests. Differences among frequencies were analyzed by the Chi-square test. Values of *p*<0.05 were considered significant for all tests.
